# Social Media Promotion of Health Tests With Potential for Overdiagnosis or Overuse: Protocol for a Content Analysis

**DOI:** 10.2196/56899

**Published:** 2024-06-04

**Authors:** Brooke Nickel, Raffael Heiss, Patti Shih, Emma Grundtvig Gram, Tessa Copp, Melody Taba, Ray Moynihan, Joshua Zadro

**Affiliations:** 1 Sydney School of Public Health Faculty of Medicine and Health The University of Sydney Sydney Australia; 2 Center for Social & Health Innovation Management Centre Innsbruk Innsbruk Austria; 3 Australian Centre for Health Engagement Evidence and Values School of Health and Society University of Wollongong Wollongong Australia; 4 Center for General Practice Department of Public Health University of Copenhagen Denmark Australia; 5 Institute for Evidence-Based Healthcare Faculty of Health Sciences and Medicine Bond University Gold Coast Australia

**Keywords:** social media, influencers, tests, overdiagnosis, overuse, evidence-based medicine, promotion

## Abstract

**Background:**

In recent years, social media have emerged as important spaces for commercial marketing of health tests, which can be used for the screening and diagnosis of otherwise generally healthy people. However, little is known about how health tests are promoted on social media, whether the information provided is accurate and balanced, and if there is transparency around conflicts of interest.

**Objective:**

This study aims to understand and quantify how social media is being used to discuss or promote health tests with the potential for overdiagnosis or overuse to generally healthy people.

**Methods:**

Content analysis of social media posts on the anti-Mullerian hormone test, whole-body magnetic resonance imaging scan, multicancer early detection, testosterone test, and gut microbe test from influential international social media accounts on Instagram and TikTok. The 5 tests have been identified as having the following criteria: (1) there are evidence-based concerns about overdiagnosis or overuse, (2) there is evidence or concerns that the results of tests do not lead to improved health outcomes for generally healthy people and may cause harm or waste, and (3) the tests are being promoted on social media to generally healthy people. English language text-only posts, images, infographics, articles, recorded videos including reels, and audio-only posts are included. Posts from accounts with <1000 followers as well as stories, live videos, and non-English posts are excluded. Using keywords related to the test, the top posts were searched and screened until there were 100 eligible posts from each platform for each test (total of 1000 posts). Data from the caption, video, and on-screen text are being summarized and extracted into a Microsoft Excel (Microsoft Corporation) spreadsheet and included in the analysis. The analysis will take a combined inductive approach when generating key themes and a deductive approach using a prespecified framework. Quantitative data will be analyzed in Stata SE (version 18.0; Stata Corp).

**Results:**

Data on Instagram and TikTok have been searched and screened. Analysis has now commenced. The findings will be disseminated via publications in peer-reviewed international medical journals and will also be presented at national and international conferences in late 2024 and 2025.

**Conclusions:**

This study will contribute to the limited evidence base on the nature of the relationship between social media and the problems of overdiagnosis and overuse of health care services. This understanding is essential to develop strategies to mitigate potential harm and plan solutions, with the aim of helping to protect members of the public from being marketed low-value tests, becoming patients unnecessarily, and taking resources away from genuine needs within the health system.

**International Registered Report Identifier (IRRID):**

DERR1-10.2196/56899

## Introduction

The idea that early detection of health conditions or diseases is always better, as it offers the best chance of being cured, has been around for decades, and continues to grow in popularity [1]. However, evidence demonstrates that lay people often overestimate the benefits and underestimate the harms of tests [2], and there is increasing evidence that inappropriate testing can harm healthy people, and the quest for early detection can lead to overdiagnosis [3-9]. Overdiagnosis is now widely recognized and occurs when generally healthy people are diagnosed or labeled with a disease that would never cause them harm [10,11]. This can occur as a result of undergoing screening tests (eg, cancer screening [12]) and can lead to the overuse of further tests and overtreatment. More recently, there have been concerns that direct-to-consumer (DTC) tests can lead to overdiagnosis of generally healthy people and overuse [13,14]. The harmful consequences of overdiagnosis can include physical harm from unnecessary tests and treatments, psychological harm from being labeled with a condition or disease that would not cause harm, and receiving invasive treatments that also carry financial consequences [11]. It can also lead to an unsustainable burden on the health care system. A review of relevant literature has identified potential drivers of overdiagnosis across 5 domains: culture, the health system, industry, professionals, and patients and the public [15]. The review found many causes, including common beliefs that “more is better,” systemic financial incentives to deliver more tests and treatments, technological changes enabling increasingly sensitive tests, and patient expectations that clinicians will “do something” [15]. While the use of more sensitive tests, the promotion of tests, and advertising and traditional media were identified as drivers of overdiagnosis, there are as yet few data on how social media may drive overdiagnosis or overuse.

In recent years, social media have emerged as an important space for commercial marketing of health products [16], including various early detection tests [17], which can be used for the screening and diagnosis of otherwise healthy people. Companies themselves are very active on social media, promoting their products through traditional advertisements that appear in users’ news feeds. Companies are also partnering with social media influencers [18]—individuals who amass large followings on social media and exert significant influence over their audience through engaging content [19]. While platforms themselves claim to regulate health information and misinformation, the current regulations around what can be promoted on social media are minimal [13]. Influencers may share health information to their audiences, even though they may not necessarily be qualified to give health information. The social media promotion of health products can often be based on personal anecdotes and opinions or, at worst, on pseudoscience or conspiracy theories. For example, studies now have identified the impact that influential social media has on health misinformation across various conditions [20-23], including most recently widespread COVID-19 misinformation [24,25] that has negatively affected people’s health behaviors [26,27]. However, little is known about how health tests are promoted on social media, whether the information provided is accurate and balanced, and if there is transparency around conflicts of interest.

This study therefore aims to quantify and understand how social media are being used to discuss or promote health tests with the potential for overdiagnosis or overuse to generally healthy people. The study will explore how the benefits and harms including overdiagnosis and overuse of the tests are discussed, whether evidence is being used in the promotion of tests, what is the overall tone, transparency around potential conflicts of interest, and what themes are being used in the discussion or promotion of tests.

## Methods

### Study Design

This study uses content analysis of information on tests from influential social media platforms, Instagram and TikTok. Content analysis [28] is a widely used qualitative research technique, which also uses quantitative methods to analyze written content, enabling themes, meanings, and concepts to be quantified and evaluated through coding. The study will be reported according to the Standards for Reporting Qualitative Research reporting guideline [29].

### Inclusion and Exclusion Criteria

The inclusion criterion includes English-language social media posts on 5 specific tests from influential international social media accounts. The 5 tests have been identified as meeting the following criteria: (1) there are evidence-based concerns about overdiagnosis or overuse, (2) there is evidence or concerns that the results of tests do not lead to improved health outcomes for generally healthy people and may cause harm or waste, and (3) the tests are being promoted on social media to generally healthy people. The authors also have interest and expertise in several of the identified tests [14,30,31].

Influential accounts are defined for the study as an individual or company account with >1000 followers that discusses the specific test in question and categorized as nano (1000-10,000 followers), micro (10,000-100,000 followers), macro (100,000-1 million followers), and mega (1 million+ followers) [32] based on number of followers. Text-only posts, images, infographics, articles, recorded videos including reels, and audio-only posts will be included. Posts from accounts with <1000 followers as well as stories, live videos, and non-English posts are excluded.

### Data Collection

The tests include the anti-Mullerian hormone (AMH) test, whole-body magnetic resonance imaging (MRI) scan, multicancer early detection (MCED) test, testosterone test, and gut microbiome test. These tests are intended for individuals of varying sociodemographic characteristics including sex and age and included a range of costs.

We chose to focus on the platforms Instagram and TikTok as they are 2 of the most fastest-growing platforms across all age demographics relevant for the promotion of the 5 identified tests. These platforms are predominantly for short-form content (eg, infographics, reels, and TikToks), which has become more popular than longer-form content (eg, long videos, long posts, and blogs) in recent years. Shorter content is likely to be more impactful as it is more accessible and receives more engagement [33]. Furthermore, these platforms have the recent documented instances of influential celebrities promoting tests with significant evidence-based concerns about overdiagnosis (eg, Kim Kardashian and the whole-body MRI on Instagram [17]).

We created new Instagram and TikTok accounts and searched the 2 platforms using keywords related to each of the tests. Keywords for each test were included based on pilot testing. We searched and screened the top posts (as defined by the platform) until we had 100 eligible posts in each platform for each test. We included and assessed posts based on the platform they have been posted on (ie, if a post was created on TikTok but posted on Instagram, then we will include it in the eligible Instagram posts), and any duplicate posts across the keywords and platforms were removed. This method was first piloted with 1 test (AMH test). Searching and screening were conducted on the 2 platforms by 1 researcher (BN) using the newly created accounts. Eligibility of posts was confirmed by a second researcher (JZ). The eligible posts were saved in the account and returned to by the researchers to summarize, extract, and analyze the data.

Summarized data on publicly available demographics (eg, date of the post), credentials of influencers or credibility of influential pages (eg, expertise—a medical doctor or not), paid partnership or disclosure (present or absence of the information), amount of followers (micro, macro, midtier, and mega), and engagement metrics (eg, views, likes, and comments) were collected.

### Included Tests

#### AMH Test for Fertility

As the average age of mothers at first birth is increasing in high-income countries [34], there has been growing attention around the AMH test, often termed the “egg timer test.” AMH test is a blood test that is used to estimate ovarian reserve; in other words, the number of eggs in a woman’s ovaries [35,36]. While AMH testing has been shown to be useful in the context of a fertility treatment [37], there is no evidence to support the AMH test as a reliable measure of fertility for women in the general population as it cannot reliably predict the likelihood of pregnancy, timing to pregnancy, or specific age of menopause for individuals [38-40]. As a result of this evidence, the American College of Obstetricians and Gynecologists strongly discourages AMH testing in women not undergoing in vitro fertilization [41]. Yet, despite clear evidence of its lack of benefit, recent data suggest that some women are taking the test as they believe it can inform their chance of conceiving [42]. Furthermore, recent content analyses of both fertility clinics’ [30] and companies’ [31] websites found that these tests are being widely marketed to the general population on the web and that many of the websites are making false and misleading claims to women about what the AMH test can tell them. Together this raises concerns about the widespread overuse of the AMH test [42].

#### Whole-Body MRI Scan for the Detection of a Range of Diseases in Their Earliest Stages (eg, Cancers)

Whole-body MRIs use strong magnetic fields and radio waves to scan and generate detailed images of the entire body. Whole-body MRI scanning has been available for more than a decade and is largely used for screening those with a high genetic risk of cancer [43]. This procedure now typically takes an hour, and despite the claimed usefulness of whole-body MRI for cancer detection in high-risk individuals, its promotion to generally healthy people in the general population is raising concerns about overdiagnosis [44,45]. There is currently no evidence that these highly sensitive tests provide net benefit for people at average risk of disease [46,47]. Alongside the potential for overdiagnosis across a number of conditions and potentially unnecessary invasive treatments, the associated anxiety and cost (ranging between approximately US $2000-US $4000) of whole-body MRIs are important to consider. Whole-body MRIs are not recommended by major international medical professional societies for people without symptoms.

#### MCED Tests

MCED tests are a type of “liquid biopsy” that aims to detect cancers early before symptoms develop. They use genomic profiling to detect cancer DNA cells circulating in the blood [48]. In 2016, the Food and Drug Administration (FDA) first approved liquid biopsies for the detection of gene mutations in circulating tumor DNA, designed for clinicians to monitor patients with cancer [49]. At present, the MCED test has not been fully approved by the FDA, yet, it has granted “breakthrough device designation” to at least 3 MCED tests. Companies are currently offering the tests to consumers and clinicians as laboratory-developed tests. While clinical and community interest continues to grow, with MCED tests being discussed as the “holy grail” for cancer detection [50], it is still unclear whether the benefits outweigh the harms. Although MCED tests have been shown in early small-scale studies to increase cancer detection and are designed to have high specificity, that is, reduce false-positive results [51-54], there are valid concerns surrounding overdiagnosis [55]. There are clinical trials [56,57] underway internationally to assess the performance and use of the tests. However, data from these trials could take over a decade, and to date, no evidence exists to robustly inform decisions about effectiveness, including reduction in late-stage cancer incidence and overall mortality. Furthermore, like the whole-body MRI scan, the cost of these tests is expensive (currently approximately US $1000), raising additional health inequity issues for population screening.

#### Testosterone Test for Low Testosterone

As men age, testosterone levels naturally begin to decline. While testosterone deficiency can be a serious medical condition in some men and requires treatment for many men, low testosterone or “low T” is a prime example of disease mongering [58,59]. Since early 2000, there has been an increase in testosterone testing and prescribing [60-62]. “Low T” awareness campaigns targeting middle-aged men in high-income countries with messages about checking testosterone levels if they had low libido, were experiencing mood changes, or had gained weight have in part driven this increase in testing and prescribing [63,64]. More recently, testing has been promoted among men in the fitness industry as a way to go on testosterone therapy and help build muscle. Testing for testosterone levels typically requires a blood sample to be taken in the morning by a medical professional; however, DTC tests that usually involve collecting a blood sample now exist, and results can be provided within days. While there is variability in guidelines of what is considered low testosterone, there is currently no evidence that testosterone testing provides clinical benefit for asymptomatic healthy men [65]. Furthermore, as testosterone levels vary, a single measurement is unreliable, and without a clear context of the person being tested, in the case of DTC testosterone testing, it runs the risk of both false-positive and false-negative results. It is also important to note that the long-term safety in relation to adverse cardiovascular events and mortality of testosterone therapy has not yet been established, with an FDA-mandated study ongoing [66].

#### Gut Microbiome Test

The gut microbiome test measures the microorganisms in a person’s gastrointestinal tract. A sample of stool is taken and sent to a laboratory and analyzed for, in some cases, hundreds of different types of bacteria, viruses, and fungi. Although the microbiome test has not been rigorously tested for accuracy or safety, with the FDA having yet to approve home microbiome tests, a growing number of companies are offering this “wellness” test with the promise of identification of diseases and disorders (and precursors to diseases and disorders) such as bowel diseases or disorders, depression, diabetes, and cancer. These tests also provide personalized reports that suggest dietary adjustments. Experts have noted that while the test looks promising, the evidence behind the claims of what the test can do is still in its infancy—with promises being made that are greater than what the current science can offer [67]. At this time, the tests can only really satisfy a person’s curiosity rather than add any value to clinical decision-making [68], which in turn can lead to overdiagnosis and overtreatment of those taking the test [69].

### Data Analysis

Data from the caption, audio or video, and on-screen text (if applicable) will be summarized and extracted into a Microsoft Excel (Microsoft Corporation) spreadsheet and included in the analysis. The analysis will take both an inductive approach when generating key themes arising from the posts and a deductive approach using a prespecified framework in line with our aims to examine (1) benefits, (2) harms including overdiagnosis and overuse, (3) evidence, (4) overall tone, and (5) financial disclosures. Independent extraction and review of the data was undertaken by 1 researcher (RM) to develop an initial list of recurring themes. The coding tool was then informed by analysis of other coding tools used in similar previous work on media reporting of new tests [70,71]. Two researchers then independently applied this coding tool to 20% (n=40 per test) of posts to evaluate the reliability of the coding tool. The level of agreement between the 2 coders across the codes (eg, benefits, harms, evidence, tone, and disclosures), and by test, will be assessed using Cohen κ and be interpreted as <0.00=poor, 0.00-0.20=slight, 0.21-0.40=fair, 0.41-0.60= moderate, 0.61-0.80=substantial, and ≥0.81=almost perfect [72]. The agreement will be considered acceptable if κ>0.6 for a sample of at least 20% (n=40) of posts per test. In some cases, the level of agreement might appear low using the κ statistic due to the high prevalence of a particular value for a variable. This is known as the κ paradox [73-76]. Therefore, we will also consider the level of agreement acceptable if κ is ≤0.6, but crude agreement is ≥85%. If κ is ≤0.6, and crude agreement is <85%, data will be recoded (using n=40, 20% blocks for each test) until an acceptable level of agreement is met. Once agreement is acceptable, 1 researcher with experience in public health and overdiagnosis will code the remaining posts.

Differences in outcomes by test and by platform, followers, and length of posts (across all 1000 posts) will be reported. Logistic regression analyses will be used to investigate whether posts by medical doctors, posts with evidence, and posts with clear financial disclosures are more or less likely to be balanced in their discussion or promotion of test in terms of benefits, harms, and overall tone. Our hypothesis is that posts by medical doctors and posts with evidence are more likely to be balanced, and posts with clear financial disclosures are less likely to be balanced. Quantitative data will be analyzed in Microsoft Excel and Stata SE (version 18.0; Stata Corp).

### Ethical Considerations

The data collected and analyzed in this study is unequivocally public. Data will be largely reported in aggregate form, however, in cases where specific excerpts (eg, quotes) are reported as examples, as is typical with the content analysis method [28], these will be short (eg, a few lines) and nonidentifiable (eg, no personal or professional information or reference will be given). A waiver of consent from The University of Sydney Human Research Ethics Committee has been granted (2023/913).

## Results

### Progress to Date

Data on each of the 5 tests have been searched and screened on Instagram and TikTok. Analysis is currently being conducted.

### Dissemination

The findings will be disseminated via publications in peer-review international medical journals and presentations at national and international conferences in late 2024 and 2025.

## Discussion

### Anticipated Findings

It is anticipated that this study will reveal fresh insights into how balanced the information and promotion of tests with the potential for overdiagnosis or overuse is on social media. This study will therefore contribute to the limited evidence base on the nature of the relationship between social media and the problems of overdiagnosis and overuse of health care services. Analyzing how these 5 popular tests are currently being promoted on 2 influential social media platforms will provide a snapshot of the wider issue.

It is now acknowledged that the public and patients alike are turning to social media for health information [77]. Therefore, the concerns driving this analysis are not that these tests exist, are being promoted on social media, or are used by those with serious symptoms, but that the tests may be being promoted to generally healthy individuals without good evidence of benefit, explicit information about their harms, and potentially relevant information on conflicts of interest [15]. Future research using similar methods can also be conducted to investigate the promotion of drugs, treatments, and disease definitions and the potential ways to reduce the volume of misleading marketing, to further add to the much-needed evidence base, as social media continues to disseminate health information and market health products.

This study has both strengths and limitations. This is the first study to analyze the promotion of tests on social media that have concerns relating to overdiagnosis or overuse. The study will only include and analyze posts on Instagram and TikTok. However, as stated in the Methods section, these were chosen as they are 2 of the most widely used and fastest-growing platforms across relevant age groups in which the tests relate to, are predominantly used for shorter more impactful content [33], and have been recently used to promote these tests by influential celebrities [17]. Posts on these platforms have been screened systematically using the methods described earlier, and the top 100 eligible posts mentioning the tests were included. Comments and replies will not be analyzed nor will posts from influencers with <1000 followers, which may have provided additional information to the analysis although it is not anticipated that this will impact the overall findings. Finally, the posts will be screened and returned to for analysis. In that time, the content may be removed, or the number of followers may have changed. Again, it is not anticipated that this will impact the overall findings.

### Conclusions

Understanding whether there is a problem in how tests with potential for overdiagnosis or overuse are discussed and promoted on social media, and the extent of the promotion, is essential to develop strategies to mitigate potential harm and plan solutions. This information will also potentially help to protect members of the public from being marketed low-value tests and becoming patients unnecessarily. It will also help to minimize overuse, which takes resources from genuine need, threatening the sustainability of health systems.

## Abbreviations

AMH: anti-Mullerian hormone
DTC: direct-to-consumer
FDA: Food and Drug Administration
MCED: multicancer early detection
MRI: magnetic resonance imaging

## Data Availability

The data sets generated and analyzed during this study are available from the corresponding author on reasonable request.
